# Assessment of Genetic Diversity and Population Structure of the Endangered *Astragalus exscapus* subsp. *transsilvanicus* through DNA-Based Molecular Markers

**DOI:** 10.3390/plants10122732

**Published:** 2021-12-11

**Authors:** Katalin Szabo, Doru Pamfil, Alexandru Sabin Bădărău, Monica Hârţa

**Affiliations:** 1 Faculty of Food Science and Technology, University of Agricultural Sciences and Veterinary Medicine Cluj-Napoca, Mănăştur St. 3-5, 400372 Cluj-Napoca, Romania; katalin.szabo@usamvcluj.ro; 2 Romanian Academy, Cluj-Napoca Branch, Republicii St. 9, 400015 Cluj-Napoca, Romania; dpamfil@usamvcluj.ro; 3 Research Centre BIOCERA, Faculty of Horticulture, University of Agricultural Sciences and Veterinary Medicine Cluj-Napoca, Mănăştur St. 3-5, 400372 Cluj-Napoca, Romania; 4 Department of Environmental Science, Faculty of Environmental Science and Engineering, Babeş-Bolyai University, 400294 Cluj-Napoca, Romania; alexandru.badarau@ubbcluj.ro

**Keywords:** conservation, genetic differentiation, habitat specificity, rarity, relict species

## Abstract

*Astragalus exscapus* L. subsp. *transsilvanicus* (Schur) Nyár. (*Fabaceae*) is a rare plant endemic to the Transylvanian Plateau, represented by 24 identified populations. Limited information on the genetic variation and population structure is available, which obstructs efficient measures for conservation strategy. The present study aimed to analyze the genetic diversity and population structure of eight populations of *A. exscapus* subsp. *transsilvanicus* revealed by sequence-related amplified polymorphism (SRAP) markers. A total of 164 bands were amplified, 96.7% of which (159) were polymorphic. Nei’s gene diversity index (He) was estimated to be 0.228 at the population level and 0.272 at the subspecies level. The genetic differentiation among populations (Gst) was 0.165, which indicated a low proportion of total genetic diversity. The analysis of molecular variance (AMOVA) indicated that 17% of the total variation of *A. exscapus* subsp. *transsilvanicus* is found among the populations, while 83% was found within the populations. A UPGMA dendrogram, principal coordinate analysis, and the STRUCTURE software grouped the populations into two clusters uncorrelated with the provenience of the 125 individuals, which might be attributed to fragmentation processes, insect pollination, population size, and specific environmental conditions of the habitats.

## 1. Introduction

Stemless milkvetch (*Astragalus exscapus* L.) is a perennial herb native to dry grasslands of Central Europe. It has small populations and a relatively high habitat specificity; therefore, it is framed a special rarity type according to Rabinowitz [1]. The species is diploid (2*n* = 16) and self-compatible; however, it requires pollination by insects, as, without pollinators, the flowers do not produce seeds, indicating a self-compatible breeding system dependent on insect pollination [2]. *A. exscapus* plants have a reduced stem, and their lemon-yellow odoriferous flowers appear in middle May gathered in racemes. The species has a swivel root system, which plays an important role in preventing soil erosion. Considered as a relict species of the Pleistocene steppe vegetation, *A. exscapus* is threatened with extinction in many countries in Europe because of pollen limitation and inbreeding depression [2]. Moreover, the species rarity can be explained partly by the low dispersal potential of seeds as the flowers are situated a few centimeters above the ground [3]. According to the Red List of endangered plant species from the Carpathians, *A. exscapus* demands sustainable conservation strategies to overcome the risk of extinction [4]. Furthermore, the endangered butterfly species *Plebejus sephirus* Kovacsi Szabo (Hungarian Zephyr Blue) is closely linked to the ecology of *A. exscapus*, given that it constitutes the predominant food-plant of the butterfly’s larval stage [5].

Interestingly, the root system of the plant under study is very similar to that of *Astragalus membranaceus* Moench, which is a consecrated medicinal plant originated in Asia, with a high content of polysaccharides [6,7], saponins [8,9,10], and isoflavonoids [11], having great implications for personalized medicine. Consequently, *A. exscapus* could provide similar bioactive abilities, health effects, and/or curative properties (under study).

Broadly, *A. exscapus* presents high interest for bio-conservational purposes, and it can be considered important from the pharmaceutic, economic, and ecologic perspectives. One of the known subspecies of *A. exscapus* is endemic to Transylvania, Romania, namely, *Astragalus exscapus* L. subsp. *transsilvanicus* (Schur) Nyár., identified in small and isolated populations of the Transylvanian Plateau. Presently, 24 populations of *A. exscapus* subsp. *transsilvanicus* have been recorded, and most studies of the subspecies focused on the ecological significance and biological traits [12,13], whereas information on its genetic diversity and population structure has not yet been reported.

Earlier studies regarding *Astragalus sericeocanus* Gontsch., an endangered species endemic to the region of Baikal lake, used inter simple sequence repeats (ISSRs) as molecular markers to evaluate the genetic diversity and population structure of the species, aimed at developing appropriate management strategies directed toward species conservation [14]. *Astragalus nitidiflorus* Jiménez Mun. et Pau, a critically endangered endemism of Murcia province, Spain, was investigated for future conservation strategies using molecular markers [15]. *Astragalus subrecognitus* Bagheri, Maassoumi & F.Ghahrem., a very rare and narrow endemic species native to the northwestern region of Iran, was analyzed using ISSR molecular markers and sequence-related amplified polymorphism (SRAP) to determine the genetic structure and the conservation status of the plant [16].

To accomplish genetic analysis of *A. exscapus* subsp. *transsilvanicus* populations, SRAP molecular markers [17] were selected, as many recent studies reflected the suitability of this technique [18,19]. SRAP markers specifically amplify polymorphic junction fragments between exons and flanking DNA, providing the level of polymorphism needed for an efficient marker system [20]. Due to its technical and economic advantages, this approach has been used in specific research areas of plant biology such as evaluation of genetic fidelity or variability [21,22,23], hybridization at different taxonomic levels [24,25], linkage analysis [26], systematics, conservation genetics, and ecology [27,28,29,30]. An extensive literature overview regarding descriptive statistics of SRAP markers with relevant number of case studies involved, encompassing 171 publications, suggested that SRAP markers should be employed for research addressing hypotheses in plant systematics, conservation, ecology, biogeography, and beyond. The review’s conclusion was that SRAP markers provide an easy-to-use, highly variable molecular marker with inherent biological significance [18].

In this study, SRAP markers were employed to assess (i) the levels of genetic diversity within and among eight investigated *A. exscapus* subsp. *transsilvanicus* populations, (ii) the degree of genetic variation among populations, and (iii) the genetic relationships and population structure of 125 individuals analyzed from eight populations of *A. exscapus* subsp. *transsilvanicus* in order to recommend conservation measures for the efficient germplasm management of this endemic species.

## 2. Results

### 2.1. Morphologic Description of Eight Populations of A. exscapus subsp. transsilvanicus

Several morphologic characteristics of the subspecies were measured in the field during sample collection for the genetic analysis. Traits such as plant height, the number of leaves per plant, the number of flowers per plant, and the inflorescences’ diameter were evaluated for 125 individuals from eight different locations, and the results are presented in Table 1 as mean values ± standard error and range. An important finding of this study was the wide variation of the average number of flowers in inflorescence within populations, with a minimum mean value recorded for individuals from Gădălin (22.3 ± 16.4) and the maximum value recorded for individuals from Săbed (76.8 ± 60.0).

**Figure 1:**
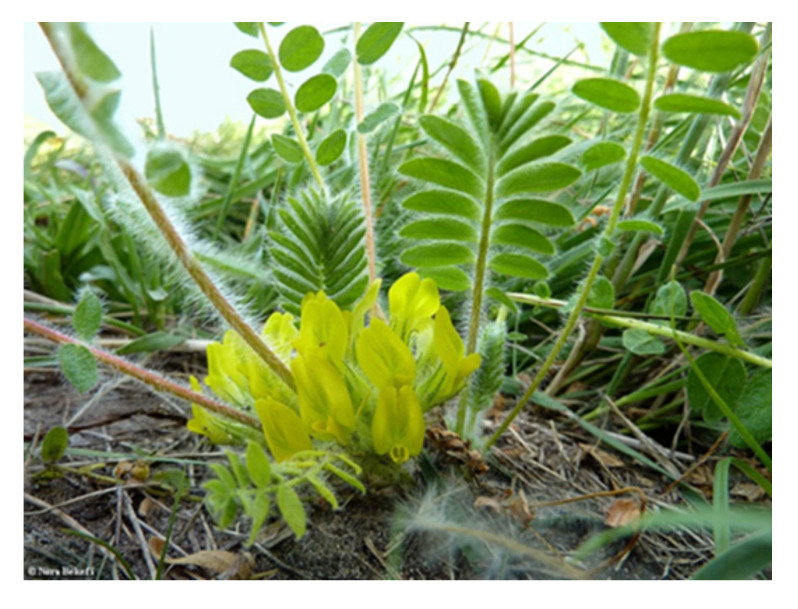
Original image of *Astragalus exscapus* L. subsp. *transsilvanicus* (Schur) Nyár. plant from the Gădălin population, Cluj County, Romania.

A representative individual from the Gădălin population is presented in Figure 1.

### 2.2. SRAP Marker Polymorphism

Fourteen SRAP primer combinations presented clear, highly polymorphic patterns and generated a total number of 164 bands with an average of 11.71 bands per primer set. The levels of polymorphism and the size of the range of PCR products amplified by SRAP primers are presented in Table 2.

The highest number of total bands (16 bands) was attained using the primer combinations Me4/Em5 and Me6/Em1 and the lowest number (nine bands) was attained using the primer combinations Me2/Em6 and Me8/Em3. The number of polymorphic bands detected by each primer combination ranged from eight (Me2/Em6) to 16 (Me6/Em1, Me4/Em5), with a mean value of 11.36 polymorphic bands. The primer average polymorphic information content (PIC) was 0.34, with values ranging between 0.23 for Me2/Em6 and 0.48 for Me4/Em4 (Table 2). These results show that the selected SRAP markers were able to assess the genetic relationships among *Astragalus exscapus* subsp. *transsilvanicus* samples collected from eight populations located on the Transylvanian Plateau. For dominant markers, such as SRAP markers, the PIC values ranged from 0 to 0.5, where 0 indicates the fixation of one allele and 0.5 denotes equal frequencies of alleles [25].

An example of an electrophoretic profile generated using the combination of the primers Me4 and Em5 is shown in Supplementary Materials Figure S1.

### 2.3. Genetic Diversity Analysis

In order to investigate the levels of genetic diversity within and among *A. exscapus* subsp. *transsilvanicus* populations, the polymorphic band percentage (PBP %), number of different alleles (Na), number of effective alleles (Ne), Shannon’s information index (I), and expected heterozygosity (He), which is also called Nei’s gene diversity, were calculated. The results of the evaluated genetic diversity indices are presented in Table 3.

As shown in Table 3, the average polymorphic band percentage (PBP) among the eight investigated populations ranged from 72.22% in the Beleni population from Cluj County to 85.42% in the Silivașu de câmpie population from Bistrita-Năsăud County. The average number of different alleles (Na) ranged from 1.493 for the Beleni (CJ) population to 1.764 for the Silivașu de câmpie (BN) population.

The Gădălin (G) population recorded the highest number of effective alleles (1.403) and the highest Shannon index (0.382). Furthermore, it presented an expected heterozygosity value of 0.247 and a polymorphic percentage of 85.42%.

The Beleni population recorded the lowest number of effective alleles (1.349), and Shannon index (0.324), and expected heterozygosity values (0.247).

Both molecular variance analysis (AMOVA) and pairwise population differentiation (PhiPT) were performed to investigate the distribution of genetic diversity within and among populations.

The total genetic diversity at the species level (Ht) was 0.2727, while the average genetic diversity within populations (Hs) evaluated by SRAP markers was 0.2279. The coefficient of genetic differentiation among populations (Gst) was 0.1645, which indicated that approximately 17% of the total genetic diversity was distributed among *A. exscapus* subsp. *transsilvanicus* populations according to POPGENE results.

Furthermore, analysis of molecular variance analysis (AMOVA) revealed that 83% of total genetic variations were contributed by differences within *A. exscapus* subsp. *transsilvanicus* populations, which was significantly higher than that among populations (17%), as summarized in Table 4. PhiPT and the gene flow (Nm) average level between populations were 0.167 (*p* < 0.001) and 2.5395, respectively.

The pairwise PhiPT provided estimates of genetic differentiation among the populations. The highest differentiation (0.480) was observed between the Săbed and Silivaşu de câmpie populations, and the lowest (0.113) was observed between the Sărmăşel Gară and Viişoara populations (Table 5).

Moreover, the populations from Mureş County (Milăşel, Săbed, and Sărmăşel Gară) showed a higher genetic differentiation (PhiPT values 0.248 to 0.438) compared to *A. exscapus* subsp. *transsilvanicus* populations from Cluj County (Beleni, Gădălin, Viişoara, and Valea Florilor), where the pairwise population PhiPT values ranged between 0.166 and 0.264 (Table 5).

### 2.4. Cluster Analysis and PCoA

**Figure 2:**
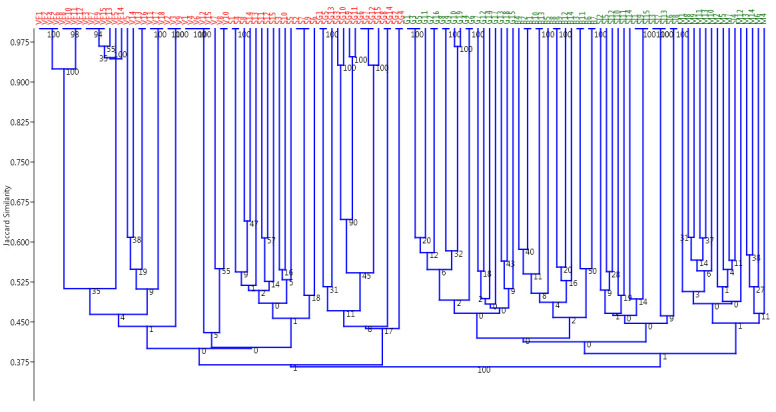
UPGMA dendrogram generated by SRAP markers, showing the relationships among *A. exscapus* subsp. *transsilvanicus* individuals and based on Jaccard’s coefficient. The two main clusters included (A, red) individuals from Valea Florilor (VF), Viişoara (V), Săbed (S), and Sărmăşel Gară (SG) populations and (B, green) individuals from Gădălin (G), Beleni (B), Silivaşu de câmpie (SI), and Milăşel (M) populations. Numbers on the branches show bootstrap values, computed from 9999 replications.

The data generated by SRAP molecular markers were used to construct a dendrogram based on the unweighted pair group method with an arithmetical average (UPGMA) algorithm (Figure 2) and Jaccard’s similarity index (Supplementary Materials Table S1). This coefficient ignores negative matches, which is appropriate in the case of dominant inherited markers where the absence of a band may be the result of different phenomena at the DNA sequence level. Moreover, SRAP markers amplified the open reading frames (ORFs) and generated the PCR patterns on the basis of the presence/absence of bands at each locus.

In this study, a high cophenetic correlation coefficient of 0.8417 between the Jaccard (J) similarity matrix and the cophenetic matrix was obtained, indicating a good fit between the built dendrogram and the similarity matrices. The UPGMA-based dendrogram grouped the *A. exscapus* subsp. *transsilvanicus* individuals into two main clusters illustrated in Figure 2. The first main cluster (Group I) contained four populations, namely, Valea Florilor (VF), Viişoara (V), Săbed (S), and Sărmăşel Gară (SG) populations. The second main cluster (Group II) consisted of Gădălin (G), Beleni (B), Silivaşu de câmpie (SI), and Milăşel (M) populations.

**Figure 3:**
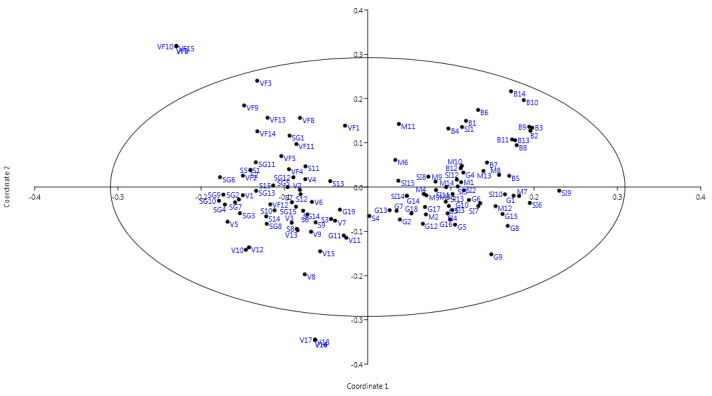
Principal coordinate analysis of eight populations of *A. exscapus* subsp. *transsilvanicus* based on SRAP molecular marker analysis.

On the other hand, the results of PCoA were largely in agreement with those obtained by the cluster analysis, further supporting the separation of individuals into two main groups, as shown in Figure 3.

One group consisted of individuals belonging to Săbed, Viişoara, Valea Florilor, and Sărmăşel Gară populations, and the other group contained the individuals from Gădălin, Milăşel, Silivaşu de câmpie, and Beleni populations.

In summary, the phylogenetic tree and PCoA showed that *A. exscapus* subsp. *transsilvanicus* populations presented different levels of genetic similarity. These findings are in accordance with the similarity levels observed in Jaccard’s similarity matrix which is presented in Supplementary Materials Table S1.

### 2.5. Population Structure Analysis

To better elucidate the relationship among the *A. exscapus* subsp. *transsilvanicus* individuals, the population structure was analyzed using STRUCTURE software. The optimal cluster value (K) of analyzed 125 individuals was 2, with the highest value of both LnP (K) and delta K recorded from the Structure Harvester v. 6.0. (Supplementary Materials Figure S2a,b).

**Figure 4:**
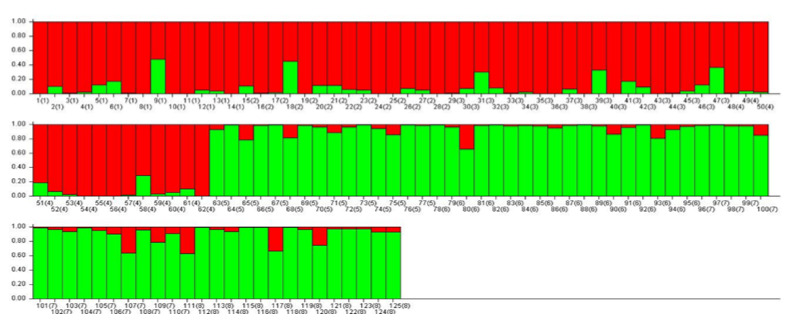
Population structure plot of 125 *A. exscapus* subsp. *transsilvanicus* individuals.

All individuals were divided into two clusters as illustrated in the bar plot of the estimated membership coefficients of each sample (Figure 4). As shown in Figure 4, each individual was represented graphically by a vertical line, and the two colors (green and red) displayed the proportion of membership (Q ˃ 0.6) of each individual to the two K clusters.

Cluster I (green) contained 59 pure individuals from Săbed, Viişoara, Valea Florilor, and Sărmăşel Gară populations, while cluster II (red) included 57 pure individuals from Milăşel, Beleni, Gădălin, and Silivaşu de câmpie populations. Individuals with admixed population assignment were from Milăşel (1), Beleni (1), Gădălin (3), Valea Florilor (2), and Viişoara (2).

## 3. Discussion

*Astragalus* is the largest plant genus in the world, including approximately 3000 species, with about one-third of existing species considered vulnerable, endangered, or critically endangered, restricted to narrow geographic and ecological regions [31].

Despite the great scientific interest in the evolution and conservation of *Astragalus* species, there are few studies on the assessment of genetic diversity through DNA-based molecular markers, and further studies on genetic diversity are necessary, particularly for endemic species [16]. In this context, the present study was conducted to better understand the genetic diversity and population structure of *Astragalus exscapus* subsp. *transsilvanicus* with the aim of providing valuable and additional information for the authorities involved in developing strategies for the sustainable conservation of local endemic species from Transylvania, Romania.

### 3.1. Effectiveness of SRAP Markers in A. exscapus subsp. transsilvanicus

Although the relatively low level of accuracy of results for estimating genetic diversity using dominant markers has generated controversial opinions, it has been well documented that SRAP is a valuable tool for detecting DNA polymorphisms and assessing genetic variation in many plant species [27,28,29]. SRAP markers are used to amplify coding regions of DNA with primers targeting open-reading frames, and they have been shown to be more robust and highly variable compared to other multilocus dominant markers such as inter simple sequence repeats (ISSRs), random amplified polymorphic DNA (RAPD), and amplified fragment length polymorphisms (AFLPs) [18].

To the best of our knowledge, there are very few reports of the use of SRAP markers in *Astragalus* species. For example, Bagheri et al. reported that SRAP proved to be an adequate and inexpensive technique to compare the genetic diversity between *Astragalus subrecognitus* Bagheri, Maassoumi & F.Ghahrem. populations from the eastern and western sides of the Qezel Ozan River in the Iranian province of Zanjan, as the nine combinations of selected primers generated 135 fragments in total, of which 93.3% were polymorphic [16]. Similar to the results previously reported by this study, SRAP markers produced reproducible and highly polymorphic fragments in the present study (164 bands in total; 99.7% polymorphic bands), which confirmed their effectiveness in assessing the genetic relationships and population structure of *Astragalus exscapus* subsp. *transsilvanicus*.

### 3.2. Genetic Diversity of A. exscapus subsp. transsilvanicus Populations

Genetic diversity in wild plant species depends on a variety of environmental factors such as life history, breeding system, seed dispersal, population size, and ecological traits that are considered mainly responsible for the level and distribution of genetic diversity within and among populations. Furthermore, to know the genetic diversity is important for the conservation of the most relevant populations [32].

In the present study, SRAP analysis of eight investigated *A. exscapus* subsp. *transsilvanicus* populations revealed a relatively high level of genetic diversity at the species level (He = 0.272, I = 0.425, PBP = 99.31%) (Table 3). Noticeable is that the mean value of expected heterozygosity at the species level (He = 0.272) was higher than the average value reported for plant species with restricted distribution (He = 0.191) [14]. Moreover, when compared with other endemic *Astragalus* species which were evaluated with different dominant markers, at the population level, Nei’s genetic diversity for *A. exscapus* subsp. *transsilvanicus* (He = 0.228) was higher compared to that of *Astragalus cremnophylax Barneby* var. *cremnophylax* (He = 0.135) [33], nearly similar to that of *Astragalus jaegerianus* Munz (0.266) [34], and lower compared to those reported for *Astragalus crassicarpus* Nutt. var. *trichocalyx* (Nutt.) Barneby (He = 0.55) [35] and *Astragalus bibullatus* Barneby & E.L.Bridges (He = 0.63) [36].

Although rare plants are usually represented by small, isolated populations spread in naturally isolated habitats, the considerable existing genetic diversity in *A. exscapus* subsp. *transsilvanicus* populations could be predominantly explained by life form and breeding system. *A. exscapus* subsp. *transsilvanicus* is considered a subspecies with a mixed breeding system; it is a self-compatible subspecies but requires pollination by insects, as, without pollinators, the flowers do not produce seeds [2]. This breeding system could explain the high level of within-species genetic diversity of *A. exscapus* and its adaptability to different environmental conditions [1]. Our results showed that *A. exscapus* subsp. *transsilvanicus* should also be able to adapt to environmental changes. As reported in previous studies [14], the breeding system of a species is a major factor in explaining levels of genetic variability at both the species and the population level, with self-crossing taxa being the least diverse and outcrossing taxa being the most diverse.

It is worth mentioning that naturally isolated habitats also provide an opportunity to examine the accumulated effects of isolation and fragmentation on the levels and distribution of genetic variation among populations. According to Travis et al., [33], habitat fragmentation and low population size have the potential to significantly affect the levels of genetic differentiation among populations over longer periods.

The proportion of genetic diversity attributed to among-population variation (Gst) has been used to estimate the genetic variation among the populations in the case of *Astragalus* species [14,15,16] using dominant inherited DNA-based molecular markers.

In the present study, the Gst of the eight populations *A. exscapus* subsp. *transsilvanicus* was 0.16, which indicated that a low proportion of total genetic diversity was observed among populations, similar to that reported for long-lived perennial species (Gst = 0.19) [37]. Moreover, the effectiveness of SRAP markers used in this study for among-population genetic diversity was confirmed by the nearly identical mean value of PhiPT (0.167) from the AMOVA analysis and the genetic differentiation coefficient Gst (0.164) from the PopGene analysis.

The AMOVA results of this study indicated that 17% of the total variation of *A. exscapus* subsp. *transsilvanicus* is found among the populations, while 83% is found within the populations. These results are in concordance with those obtained by Vicente et al. [15] in a study conducted in Spain with five populations of *Astragalus nitidiflorus* Jimenez & Pau, an endemic species threatened with extinction. Our results are also in accordance with the results obtained previously by Wall et al. [38] regarding the endangered species of *Astragalus michauxii* (Kuntze) F.J. Herm. performed using codominant SSR markers, where the percentage of interpopulation variability was determined to be 9.66% of total genetic variation.

For endemic species, maintaining the genetic diversity is very important because it helps to increase the population fitness in a changing environment [14]. Gene flow (Nm) helps to improve the level of genetic diversity of plant populations and is also an important factor affecting genetic differentiation [19]. When the value of Nm >1, there is enough gene flow to negate the effects of genetic drift. In this study, the value of gene flow (Nm = 2.54) among *A. exscapus* subsp. *transsilvanicus* populations showed that the populations are not yet affected by genetic drift. These results are consistent with those reported by Alexander et al. 2004 [39] in a study related to the genetic diversity of *Astragalus oniciformis* Barneby (Nm = 3.93) using ISSR markers.

### 3.3. Population Structure of A. exscapus subsp. transsilvanicus

The population structure of 125 *A. exscapus* subsp. *transsilvanicus* individuals evaluated by STRUCTURE software indicated two clusters (Figure 4). Among the analyzed individuals, 116 were relatively pure according to the proportion of membership Q ˃ 0.6 as the pure standard. The other nine individuals, from Milăşel (1), Beleni (1), Gădălin (3), Valea Florilor (2), and Viişoara (2), were genetically admixed.

Correspondingly, the principal coordinate analysis and UPGMA built dendrogram similarly indicated two main clusters of *A. exscapus* subsp. *transsilvanicus* individuals: individuals from Valea Florilor (VF), Viişoara (V), Săbed (S) and Sărmăşel Gară (SG) populations and individuals from Gădălin (G), Beleni (B), Silivaşu de câmpie (SI), and Milăşel (M) populations. It is noteworthy that geographically distant populations were genetically related, as revealed by both the UPGMA dendrogram and the PCoA graphical representation, based on Jaccard’s similarity coefficient.

The population structure of *A. exscapus* subsp. *transsilvanicus* was found to be influenced mainly by its geographic distribution, which might not be directly attributed to geographic distances between the eight analyzed populations.

According to previous work related to *A. exscapus* populations from central Germany, rare plant species may be limited in abundance not only due to habitat specificity, but also due to biological attributes, such as low fecundity and/or low dispersal potential [3]. *A. excapus* has a disjointed distribution in Europe, being restricted to dry regions, and it can be found on south-facing slopes with high irradiation and a pronounced summer drought period. This is also the case for *A. exscapus* subsp. *transsilvanicus*; therefore, its rarity might be partially caused by limited dispersal potential together with habitat fragmentation and grazing disturbances.

Related to the species biology and reproductive system, the plants start to flower in mid-April and finish flowering at the end of May, while the lemon-yellow odoriferous flowers appear in five to over 100 racemes, each with four to eight flowers a few centimeters above the ground [2]. According to Becker et al. (2011), *A. exscapus* is pollinated by bumblebees such as *Bombus hortorum* or *B. pascuorum*, which gather both nectar and pollen from the flowers; after successful pollination, one pod is produced per flower, which contains between one and 18 bean-shaped seeds. Considering that the species reproduces solely via seeds and that 90% of the seeds are dispersed less than 50 cm distance from the mother plant, there is clear indication of the low dispersal potential of the species, which further affects the construction of the population.

On the other hand, it is assumed that, before the fragmentation process of the habitats by human intervention, a single homogeneous population of *Astragalus exscapus* subsp. *transsilvanicus* existed, which gradually suffered a process of adaptation to the new conditions of growth and development due to the progressive isolation of the habitats [12].

### 3.4. Conservation Implications

The low level of variation among the populations and the relatively high level of intra-population variation in *Astragalus exscapus* subsp. *transsilvanicus* reflect the recent isolation of the populations, as well as the fragmentation of the species habitat (represented by well-preserved xeric to mesic grasslands from the forest–steppe of the Transylvanian Plain). Apparently, the gene flux between populations was recently interrupted, and the remaining populations are relics resulting from the fragmentation and constriction of much larger populations which were once close to another. This is of major importance for conservation principles and strategies applied to this endemic subspecies and its Natura 2000 habitat, which is also endemic to Transylvania (6240 * Sub-Pannonian Steppe Grasslands) [40].

The results of the study highlight the idea proposed by Bădărău et al. [41] that the taxon was widespread in the Transylvanian Plain and regressed to the present relic populations because of the human impact in the last two centuries. This also applies, according to the same authors, to many other species characteristic for the 6240 * habitat, such as *Serratula radiata* (Waldst. et Kit.) M. Bieberstein, *Salvia nutans* L., *Astragalus dasyanthus* Pallas, *Pontechium maculatum* (L.) Böhle et Hilger, *Crambe tataria* Sebeok, *Peucedanum ruthenicum* M. Bieberstein, and *Plantago argentea* Chaix. Their present situation is a consequence of the last two agrarian reforms in Transylvania: the first from 1856 applied in the former Habsbrug Empire, and the second from 1921 in post-World War I Romania [42]. Before the 19th century, the Transylvanian Plain was a region of forest–steppe with only a few scattered forests avoided by the population (despite its fertile soils) due to the frequent Tartarian invasions from the east, against which the only refuge was the larger forests in the rest of the Transylvanian Basin [43]. As this threat ceased to exist in the 1800s, the successive regional and statal administrations colonized the vast largely uninhabited steppe grasslands from the center of Transylvania with large numbers of people. The colonization was so intense that, despite being the area with the lowest density of population and villages of Transylvania in 1856, this area became the region with the highest density of villages across Romania, with the main occupation of the colonized farmers being sheep breeding and agriculture [44]. The intense exploitation that started almost 170 years ago dismantled the metapopulational connections for *Astragalus exscapus* subsp. *transsilvanicus* and species alike in Transylvania, while the steppe grasslands of the 6240 * habitats were destroyed by plowing or drastically degraded by sheep overgrazing.

The conservation strategies for the steppe species of the region, which were confirmed to be much more widespread in the past, should be based on ecological reconstruction of the habitat 6240 * and re-expansion of their populations in the reconstructed areas, resulting in the re-establishment of their metapopulational complexes. This is necessary because they were characteristic elements of the ancient Transylvanian forest–steppe landscape, which is intended to be reconstructed via diverse projects [45]. Studies like the present one can help separate the once common species of this landscape from species which were rare/relic due to natural causes prior to the intense human impact produced by the agrarian reforms (i.e., *Centaurea ruthenica* Lam., *Centaurea trinervia* Stepham., *Iris pontica* Zap., *Colchicum versicolor* Ker Gawl., *Nepeta ucranica* L., *Scutellaria supina* L., *Goniolimon tataricum* (L.) Boiss., etc.). For this latter category of species, different conservation principles and strategies must be applied, focused on the maintenance of the population at the local level, according to the principles of “Peripheral Populations Biology” [46,47].

## 4. Materials and Methods

### 4.1. Plant Material

**Figure 5:**
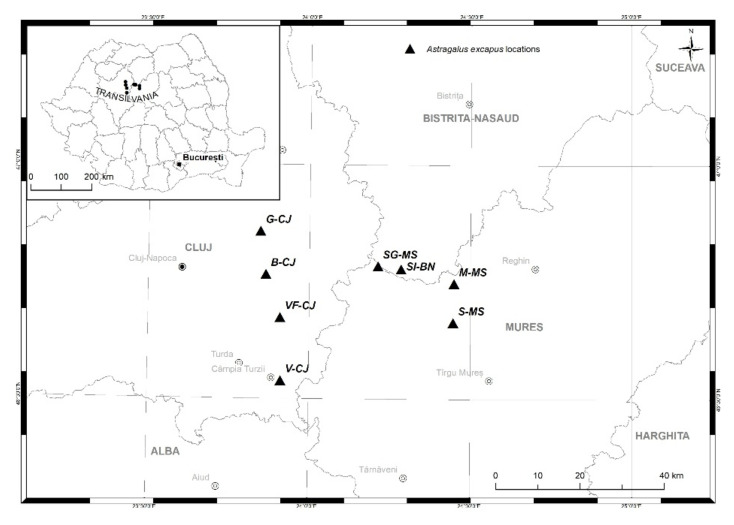
Map with the sites where samples of *A. exscapus* subsp. *transsilvanicus* were collected. B, Beleni population, Cojocna, Cluj County (CJ); V, Viișoara population; VF, Valea Florilor population; G, Gădălin population, Mureș County (MS); M, Milășel population; S, Săbed population; SG, Sărmășel Gară population; SI, Silivașu de câmpie population, Bistrița Năsăud County (BN).

The biological material was represented by 125 individuals of the subspecies *Astragalus exscapus* L. subsp. *transsilvanicus* (Schur) Nyár. Young leaf samples were collected from eight different locations shown in Figure 5, and the linear distance between the samples in the same population was not less than 50 m.

Eight populations were sampled in May 2014 representing the range of census size, area sizes, number of analyzed individuals, and geographical spread of the *A. exscapus* subspecies, as shown in Table 6. The geographical coordinates of the population sites were recorded using a global positioning system (GPS), and no specific permission was required for these locations.

### 4.2. DNA Isolation

For DNA isolation, 5–6 young leaves were collected from the plants; the samples were coded, placed into plastic zip-lock bags, and maintained at −20 °C until further examination. The genomic DNA was extracted using the CTAB-based method according to a previously published protocol [48]. DNA concentration and purity were determined using a NanoDrop-1000 spectrophotometer (Thermo Fisher Scientific, Waltham, MA, USA). Prior to SRAP analysis, DNA samples were diluted to 50 ng/µL using double-distilled water.

### 4.3. SRAP Analysis

To perform genetic analysis of *A. exscapus* subsp. *transsilvanicus* samples with SRAP markers, a primer combination screening process was assessed; eight forward and eight reverse primers (GeneriBiotech, Hradec Králové, Czechia) were tested on the basis of PCR amplification products as described in a previous study [48]. Of the 64 resulting primer combinations, 14 were used in this study to amplify all 125 samples, established according to the clarity of reproducible DNA bands and the high level of the polymorphism (Table 2).

PCR reactions were carried out in a 96-well Gradient Palm-Cycler (Corbett Research, Northampton, MA, USA) following the protocol extensively described by Li and Quiros [27]. The reaction volumes were adjusted to 15 μL for higher efficacy [25]. Each reaction consisted of 1.5 mM MgCl_2_, 0.2 mM of dNTPs, 0.3 µM of both forward and reverse primers (Kaneka-Eurogentec, Liège, Belgium), 1 U of Taq DNA polymerase (Promega, Madison, WI, USA), nuclease-free water (Sigma-Aldrich GmbH, Darmstadt, Germany), and genomic template DNA (2 µL).

The PCR temperature cycling conditions were (i) initial denaturation at 94 °C for 5 min, (ii) 5 cycles of 1 min of denaturation at 94 °C, 1 min of annealing at 35 °C, and 1 min of elongation at 75 °C, followed by 35 cycles of denaturation at 94 °C for 1 min, annealing at 50 °C for 1 min, and elongation at 72 °C for 1 min, and (iii) a final elongation step of 10 min at 72 °C. PCR amplifications were repeated twice for each primer combination to ensure the reproducibility of the results.

Separation of the amplification products was performed on 1.6% agarose gels (Promega, Madison, WI, USA) in 1× TAE at 100 V and 176 mA for 1.5–2 h. The gels were visualized in a UVP Biospectrum AC Imaging System (UVP BioImaging Systems, Hanover, Germany) after staining with 0.5 μg/mL EtBr (Sigma-Aldrich GmbH, Darmstadt, Germany) for 20 min.

### 4.4. Data Analysis

SRAP gel images were analyzed using TotalLab TL120 software (Nonlinear Dynamics, Newcastle upon Tyne, UK). The band size was determined using a 100 bp DNA step ladder (Promega, Madison, WI, USA).

The electrophoretic patterns of each primer were scored as band present (1) or band absent (0) and transferred to an original binary matrix using MS Excel. The total number of bands (TNB), number of polymorphic bands (NPB), and percentage of polymorphic bands (PPB) were counted. In order to estimate the allelic variation of each marker combination, the polymorphic information content (PIC) was calculated using the following equation:

PICi = 2fi (1 − fi),

where PICi is the polymorphic information content of marker ‘i’, ‘fi’ is the frequency of the amplified allele, and ‘1 − fi’ is the frequency of the null allele [49]. The original binary matrix that included 125 individuals and 144 generated SRAP loci was used for further data analysis.

Pairwise comparisons between individuals were used to calculate the Jaccard (1908) coefficient of genetic similarity in PAST (v 3.15) software [50]. The PAST program was also used to construct a dendrogram according to the unweighted pair group method with the arithmetic mean algorithm (UPGMA). The goodness of fit between the cophenetic matrix and the original similarity matrix was measured using the cophenetic correlation coefficient generated in the PAST program with bootstrapping number *N* = 9999.

Principal coordinate analysis (PCoA) was used to generate a coordinate graph in which the points in the Cartesian plane represent the individuals from the eight analyzed populations based on the Jaccard’s genetic similarity index and using the PAST program.

The genetic diversity indices were calculated using POPGENE1.32 software [51], i.e., the observed allele number (Na), the effective allele number (Ne), Nei’s gene diversity (He), Shannon information index (I), total genetic diversity (Ht), genetic diversity within populations (Hs), genetic differentiation coefficient (Gst), and gene flow (Nm).

Distribution of the genetic variation at two levels—within populations and among populations—based on SRAP marker patterns was determined by analysis of molecular variance (AMOVA) while presuming the Hardy–Weinberg equilibrium in the sampled populations after 999 permutations of a significance test. Furthermore, the pairwise population differentiation (PhiPT) was calculated using the GenAlEx 6.51 package [50,51].

The genetic structure of the analyzed populations was evaluated using a Bayesian model in STRUCTURE software, version 2.3.4 [52]. K (unknown) RPPs (reconstructed panmictic populations) were computed for 125 individuals and 144 loci using the recessive alleles model. Ten independent runs were conducted for each K with a burn-in period of 10,000 and 100 iterations. Delta K values were used to evaluate best-fit number of population clusters using the Structure Harvester v 6.0 online program [52].

## 5. Conclusions

The findings of this study suggest that current threats of *A. exscapus* subsp. *transsilvanicus*, including the habitat loss due to grazing disturbance, have not yet affected the genetic diversity of this species. However, there is a certain need for bio-conservation of populations to preserve the genetic integrity and the genetic diversity found in this species. Recommended conservation measures include monitoring population genetics, restoring the suitable habitats, and maintaining the effective population size.

## Figures and Tables

**Table 1:** Morphologic data of *A. exscapus* subsp. *transsilvanicus* samples recorded from eight analyzed populations.

Population	Plant Height (mm)		Number of Leaves/Plant		Number of Flowers/Plant		Inflorescence Diameter (mm)	
	Mean ± SE	Range	Mean ± SE	Range	Mean ± SE	Range	Mean ± SE	Range
M	230.9 ± 23.7	195–265	26.1 ± 18.4	9–73	44.6 ± 37.7	15–143	68.8 ± 37.1	9–140
B	158.8 ± 40.5	95–250	19.5 ± 9.4	7–45	28.8 ± 17.8	4–65	53.0 ± 16.8	19–76
G	159.6 ± 44.3	84–260	21.5 ± 14.4	5–52	22.3 ± 16.4	8–73	51.5 ± 21.9	22–105
SI	188.3 ± 49.9	93–270	17.8 ± 11.5	8–52	29.2 ± 24.1	8–89	57.8 ± 21.4	23–110
S	260.6 ± 62.8	140–323	34.3 ± 21.0	12–82	76.8 ± 60.0	3–227	102.4 ± 43.9	16–175
V	211.7 ± 59.3	120–320	22.5 ± 12.2	6–43	41.4 ± 16.3	12–75	89.4 ± 34.8	28–147
SG	163.5 ± 51.0	59–255	22.2 ± 17.9	7–62	27.8 ± 14.6	13–47	82.8 ± 47.6	28–134
VF	298.7 ± 27.1	240–332	39.5 ± 23.1	8–94	73.8 ± 52.2	13–191	100.3 ± 38.6	30–175

**Table 2:** SRAP primer sequences and analysis of the generated banding patterns of *A. exscapus* subsp. *transsilvanicus*.

SRAP Primers	Forward Primer (5′−3′)	Reverse Primer (5′−3′)	NTB	NPB	Size (bp)	PPB (%)	PIC
Me1/Em6	TGAGTCCAAACCGGATA	GACTGCGTACGAATTGCA	11	11	167–1107	100	0.42
Me2/Em1	TGAGTCCAAACCGGAGC	GACTGCGTACGAATTAAT	10	9	187–879	90	0.28
Me2/Em6	TGAGTCCAAACCGGAGC	GACTGCGTACGAATTGCA	9	8	161–1263	88.9	0.23
Me3/Em3	TGAGTCCAAACCGGAAT	GACTGCGTACGAATTGAC	10	10	211–1144	100	0.36
Me4/Em2	TGAGTCCAAACCGGACC	GACTGCGTACGAATTTGC	11	11	218–1013	100	0.40
Me4/Em4	TGAGTCCAAACCGGACC	GACTGCGTACGAATTTGA	12	12	217–910	100	0.48
Me4/Em5	TGAGTCCAAACCGGACC	TGAGTCCAAACCGGATA	16	15	236–1733	93.75	0.25
Me5/Em2	TGAGTCCAAACCGGAAG	GACTGCGTACGAATTTGC	10	9	204–857	90.00	0.26
Me5/Em6	TGAGTCCAAACCGGAAG	GACTGCGTACGAATTGCA	11	10	182–1068	90.90	0.25
Me6/Em1	TGAGTCCAAACCGGACA	GACTGCGTACGAATTAAT	16	16	237–1700	100	0.37
Me6/Em8	TGAGTCCAAACCGGACA	GACTGCGTACGAATTCAC	14	14	210–1548	100	0.31
Me8/Em2	TGAGTCCAAACCGGACT	GACTGCGTACGAATTTGC	10	10	235–1200	100	0.36
Me8/Em3	TGAGTCCAAACCGGACT	GACTGCGTACGAATTGAC	9	9	220–1373	100	0.46
Me1/Em2	TGAGTCCAAACCGGATA	GACTGCGTACGAATTTGC	15	15	175–1411	100	0.32
Total			164	159	-	-	-
Mean			11.71	11.36		96.7	0.34

NTB, number of total bands; NPB, number of polymorphic bands; bp, base pair; PPB, percentage of polymorphic bands; PIC, polymorphism information content.

**Table 3:** Genetic diversity indices of *Astragalus exscapus* subsp. * transsilvanicus* populations based on SRAP molecular marker analysis.

Population	Code	*N*	PBP (%)	Na	Ne	I	He
Milǎșel (MS)	M	14.00	75.69%	1.576	1.389	0.358	0.234
Cojocna-Dl.Beleni (CJ)	B	14.00	72.22%	1.493	1.349	0.324	0.210
Gǎdǎlin (CJ)	G	19.00	85.41%	1.743	1.403	0.382	0.247
Silivașu de câmpie (BN)	SI	15.00	85.42%	1.764	1.374	0.370	0.235
Sǎbed (MS)	S	15.00	72.92%	1.514	1.355	0.325	0.212
Viișoara (CJ)	V	18.00	78.47%	1.611	1.363	0.343	0.221
Sǎrmǎșel Garǎ (MS)	SG	15.00	78.47%	1.611	1.390	0.363	0.236
Valea Florilor (CJ)	VF	15.00	72.92%	1.528	1.373	0.349	0.227
Average		15.50	77.69%	1.605	1.374	0.352	0.228
Species-level			99.31	1.993	1.445	0.425	0.272

PBP (%) = polymorphic bands percentage, *N* = No. of samples; Na = No. of different alleles, Ne = No. of effective alleles = 1/(p^2^ + q^2^), I = Shannon’s information index = −1 × (p × ln (p) + q × ln (q)), He = expected heterozygosity = 2 × p × q.

**Table 4:** The analysis of molecular variance (AMOVA) of *Astragalus exscapus* subsp. *transsilvanicus* populations using SRAP markers.

Source of Variation	DF	SS	MS	Est. Var.	%	PhiPT
Within populations	116	2259.464	19.478	19.478	83%	
Among populations	7	558.584	79.798	3.900	17%	0.167 **
Total	123	2818.048		23.378	100%	

DF, degrees of freedom; SS, sum of squares; MS, mean square deviations; Est.Var., estimated variance component; percentage of total variance (%) contributed by each component and significance of variance (*p*-value); ** *p* ˂ 0.001.

**Table 5:** Pairwise population PhiPT values based on 999 permutations from AMOVA analysis among *Astragalus exscapus* subsp. *transsilvanicus* populations (all PhiPT values were significantly greater than 0, *p* < 0.0001).

Population	M	B	G	SI	S	V	SG	VF
M	-							
B	0.159	-						
G	0.188	0.166	-					
SI	0.199	0.164	0.158	-				
S	0.438	0.406	0.438	0.480	-			
V	0.246	0.205	0.218	0.227	0.348	-		
SG	0.248	0.214	0.211	0.233	0.361	0.113	-	
VF	0.302	0.264	0.292	0.291	0.449	0.285	0.243	-

**Table 6:** Original information and number of *A. exscapus* subsp. *transsilvanicus* samples used in this study.

Population.	Code	No. of Samples	Latitude (N)	Longitude (W)	Area (m^2^)	Census Size
Milǎșel, Mureș County	M	14	46°44′52″	24°26′55″	654	109
Cojocna, Beleni hill, Cluj County	B	15	46°45′57″	23°51′52″	1148	395
Gǎdǎlin, Cluj County	G	19	46°51′28″	23°50′50″	525	68
Silivașu de câmpie, Bistrița Năsăud County	SI	15	46°46′41″	24°17′04″	5546	1109
Săbed, Mureș County	S	15	46°39′52″	24°26′46″	1205	182
Viișoara, Cluj County	V	18	46°33′28″	23°47′48″	806	128
Sǎrmǎșel Garǎ, Mureș County	SG	15	46°47′03″	24°12′45″	463	93
Valea florilor, Cluj County	VF	15	46°40′25″	23°54′35″	3706	335

## Supplementary Materials

The following are available online at https://www.mdpi.com/article/10.3390/plants10122732/s1: Figure S1. Electrophoretic profiles of individuals from the eight studied populations, generated by the SRAP primer combination Me4/Em5; Figure S2. Graphical representation of population structure using STRUCTURE 2.3.4. software and Harvester v. 6.0 online program. (a) Evanno table output; (b) the median and variance of the estimated probability value for each K value; Table S1. Jaccard’s similarity coefficient among 125 individuals from eight populations of A. exscapus subsp. transsilvanicus based on SRAP data scored.
