# Supplementary material for: Assessment of Genetic Diversity and Population Structure of the Endangered Astragalus exscapus subsp. transsilvanicus through DNA-Based Molecular Markers
Source: Plants (Basel). 2021 Dec 11;10(12):2732. doi: 10.3390/plants10122732 (PMC8707493; doi:10.3390/plants10122732)
Supplement: Supplementary file 1 [file plants-10-02732-s001.zip › plants-1466450-supplementary/SupplementaryMaterials/Figure S1 SRAPgel_images.pdf]

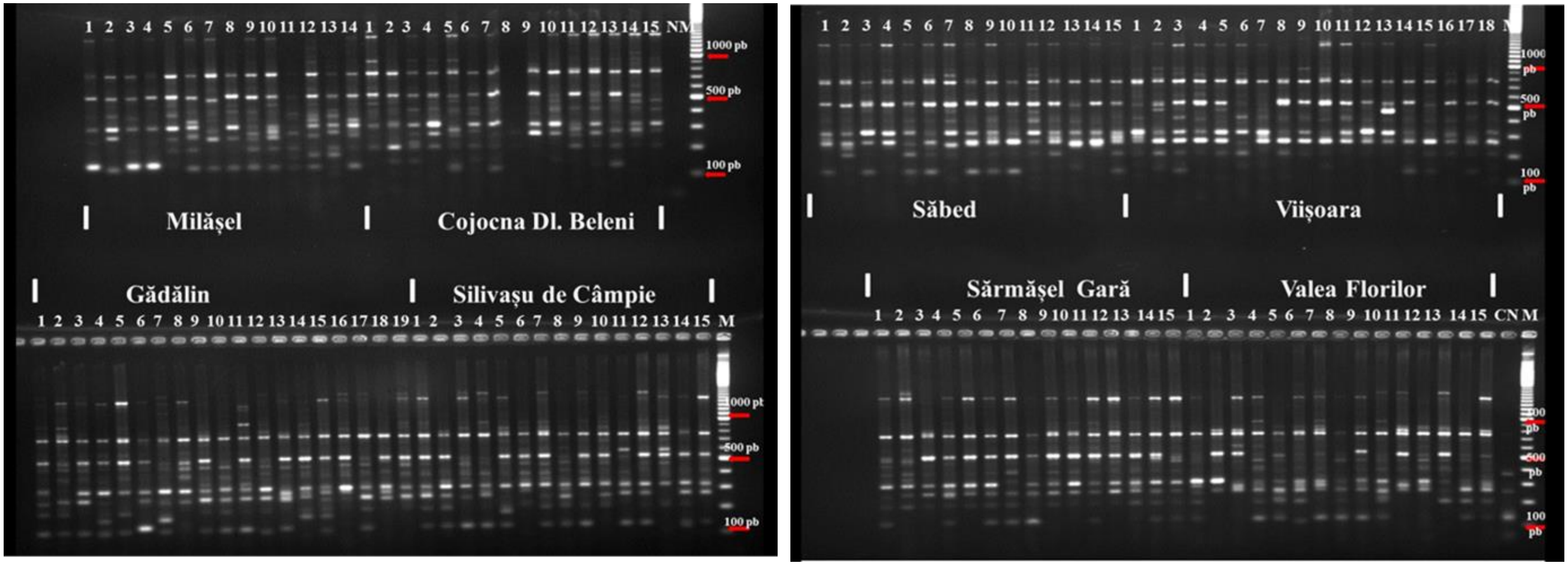

Supplementary Figure S1 Electrophoretic profiles of individuals from the eight studied populations, generated by the SRAP primer combination Me4/Em5
