# Supplementary material for: Assessment of Genetic Diversity and Population Structure of the Endangered Astragalus exscapus subsp. transsilvanicus through DNA-Based Molecular Markers
Source: Plants (Basel). 2021 Dec 11;10(12):2732. doi: 10.3390/plants10122732 (PMC8707493; doi:10.3390/plants10122732)
Supplement: Supplementary file 1 [file plants-10-02732-s001.zip › plants-1466450-supplementary/SupplementaryMaterials/Figure S2 a_b_STRUCTURE.pdf]

K	Reps	Mean LnP(K)	Stdev LnP(K)	Ln'(K)	|Ln''(K)|	Delta K
1	10	-8779.420000	13.434268	—	—	—
2	10	-8405.200000	16.375456	374.220000	233.230000	14.242657
3	10	-8264.210000	23.783395	140.990000	28.920000	1.215974
4	10	-8152.140000	41.402823	112.070000	52.880000	1.277208
5	10	-8092.950000	57.517133	59.190000	—	—

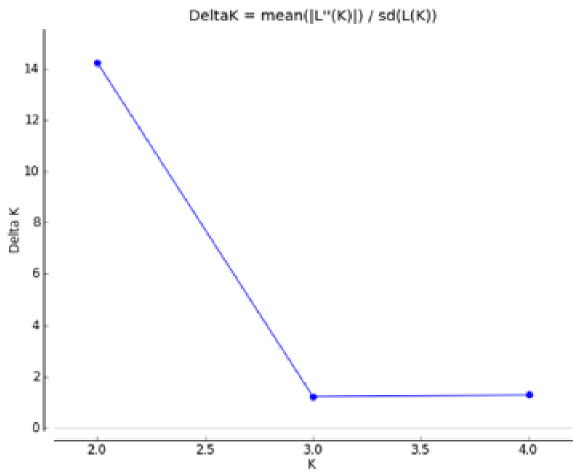

(a) (b)

Supplementary Figure S2. Graphical representation of population structure using STRUCTURE 2.3.4. software and Harvester v. 6.0 online program. (a) Evanno table output; (b) the median and variance of the estimated probability value for each K value.
